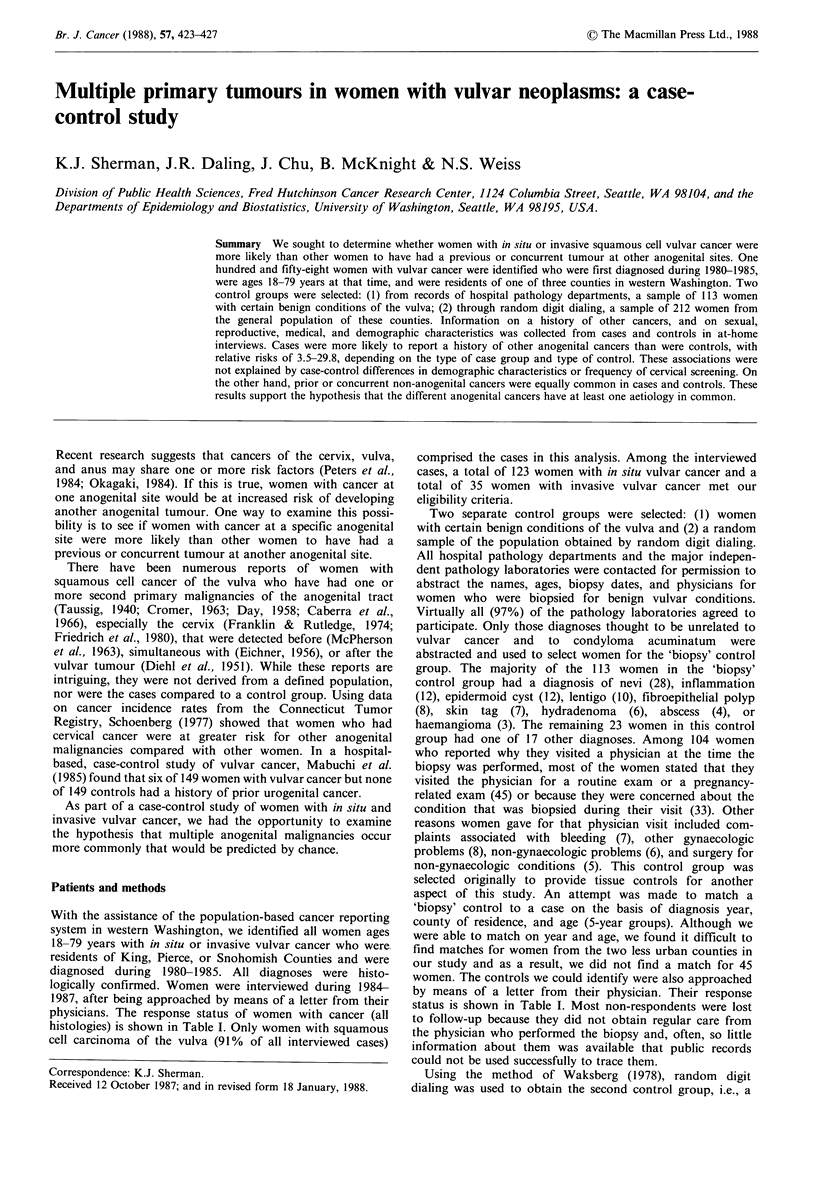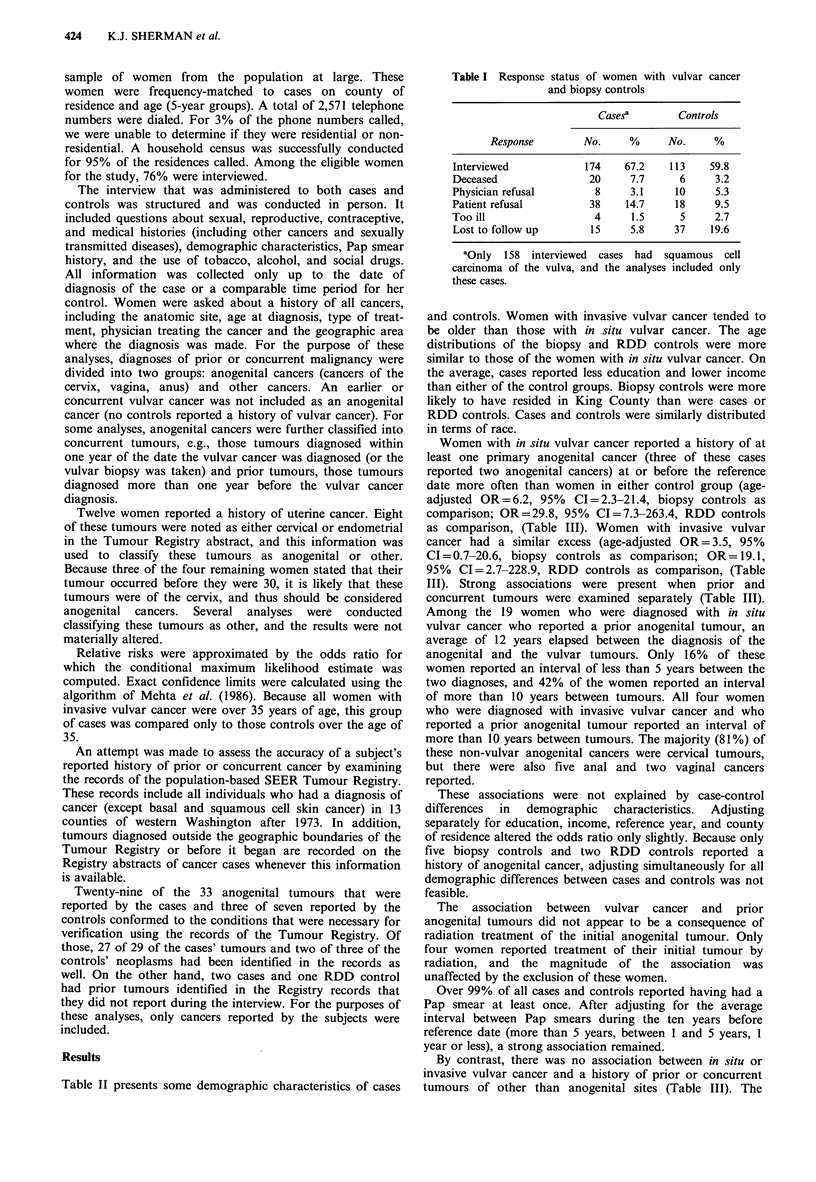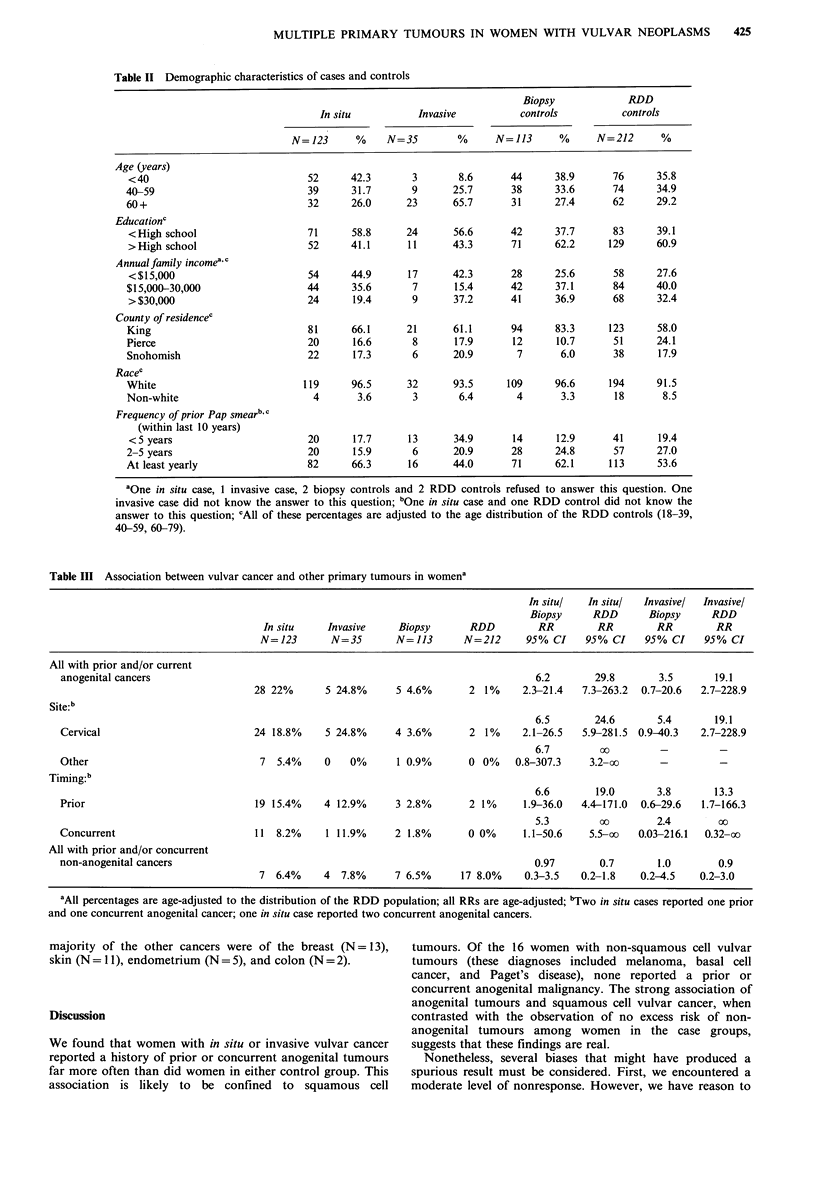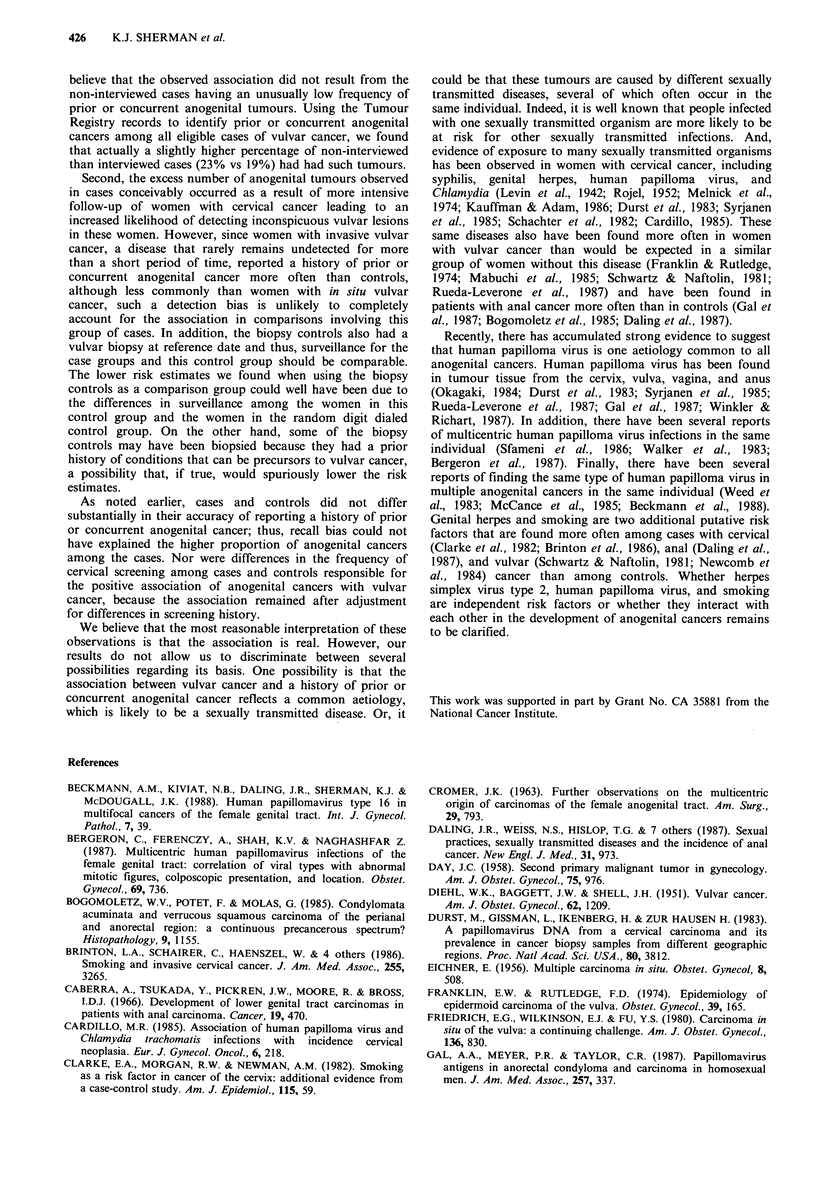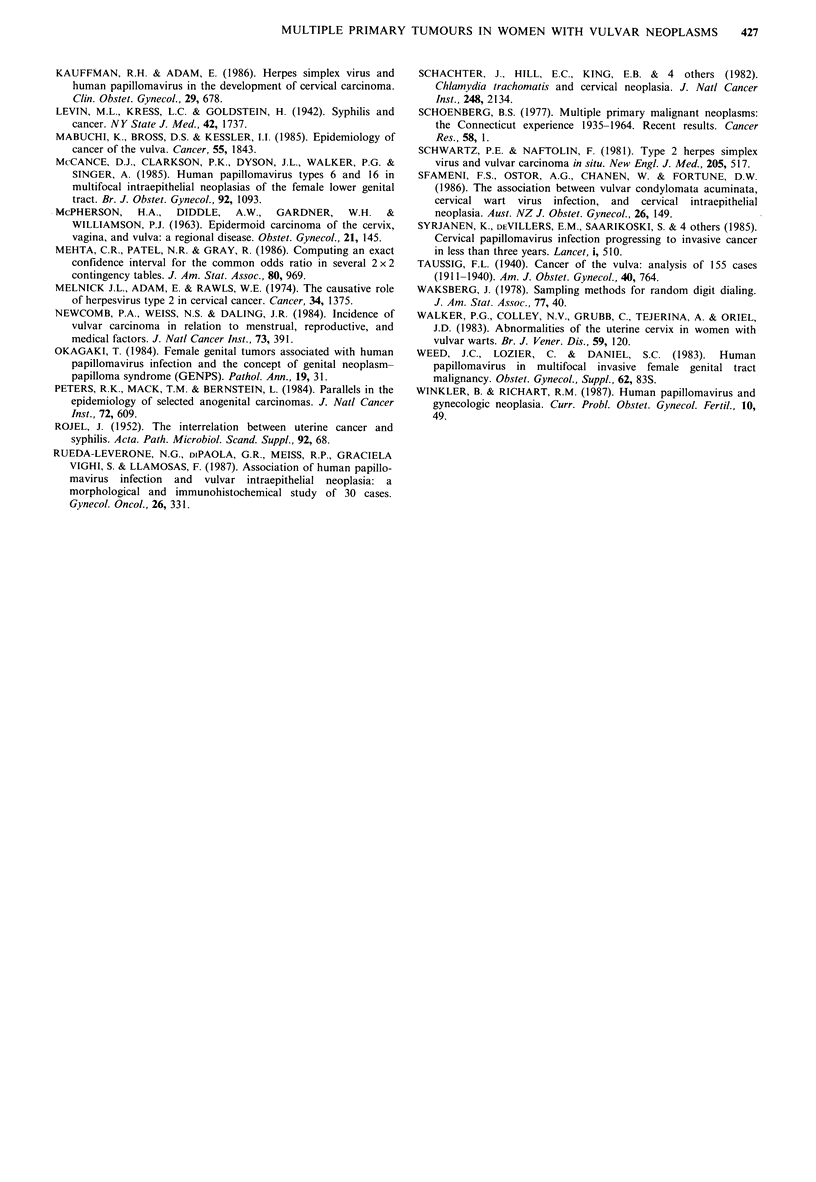# Multiple primary tumours in women with vulvar neoplasms: a case-control study.

**DOI:** 10.1038/bjc.1988.96

**Published:** 1988-04

**Authors:** K. J. Sherman, J. R. Daling, J. Chu, B. McKnight, N. S. Weiss

**Affiliations:** Division of Public Health Sciences, Fred Hutchinson Cancer Research Center, Seattle, WA 98104.

## Abstract

We sought to determine whether women with in situ or invasive squamous cell vulvar cancer were more likely than other women to have had a previous or concurrent tumour at other anogenital sites. One hundred and fifty-eight women with vulvar cancer were identified who were first diagnosed during 1980-1985, were ages 18-79 years at that time, and were residents of one of three counties in western Washington. Two control groups were selected: (1) from records of hospital pathology departments, a sample of 113 women with certain benign conditions of the vulva; (2) through random digit dialing, a sample of 212 women from the general population of these counties. Information on a history of other cancers, and on sexual, reproductive, medical, and demographic characteristics was collected from cases and controls in at-home interviews. Cases were more likely to report a history of other anogenital cancers than were controls, with relative risks of 3.5-29.8, depending on the type of case group and type of control. These associations were not explained by case-control differences in demographic characteristics or frequency of cervical screening. On the other hand, prior or concurrent non-anogenital cancers were equally common in cases and controls. These results support the hypothesis that the different anogenital cancers have at least one aetiology in common.


Br. J. Cancer (1988), 57, 423–427 © The Macmillan Press Ltd., 1988

### Multiple primary tumours in women with vulvar neoplasms: a case-control study

K.J. Sherman, J.R. Daling, J. Chu, B. McKnight & N.S. Weiss

*Division of Public Health Sciences, Fred Hutchinson Cancer Research Center, 1124 Columbia Street, Seattle, WA 98104, and the Departments of Epidemiology and Biostatistics, University of Washington, Seattle, WA 98195, USA.*

**Summary** We sought to determine whether women with *in situ* or invasive squamous cell vulvar cancer were more likely than other women to have had a previous or concurrent tumour at other anogenital sites. One hundred and fifty-eight women with vulvar cancer were identified who were first diagnosed during 1980–1985, were ages 18–79 years at that time, and were residents of one of three counties in western Washington. Two control groups were selected: (1) from records of hospital pathology departments, a sample of 113 women with certain benign conditions of the vulva; (2) through random digit dialing, a sample of 212 women from the general population of these counties. Information on a history of other cancers, and on sexual, reproductive, medical, and demographic characteristics was collected from cases and controls in at-home interviews. Cases were more likely to report a history of other anogenital cancers than were controls, with relative risks of 3.5–29.8, depending on the type of case group and type of control. These associations were not explained by case-control differences in demographic characteristics or frequency of cervical screening. On the other hand, prior or concurrent non-anogenital cancers were equally common in cases and controls. These results support the hypothesis that the different anogenital cancers have at least one aetiology in common.

Recent research suggests that cancers of the cervix, vulva, and anus may share one or more risk factors (Peters *et al.*, 1984; Okagaki, 1984). If this is true, women with cancer at one anogenital site would be at increased risk of developing another anogenital tumour. One way to examine this possibility is to see if women with cancer at a specific anogenital site were more likely than other women to have had a previous or concurrent tumour at another anogenital site.

There have been numerous reports of women with squamous cell cancer of the vulva who have had one or more second primary malignancies of the anogenital tract (Taussig, 1940; Cromer, 1963; Day, 1958; Caberra *et al.*, 1966), especially the cervix (Franklin & Rutledge, 1974; Friedrich *et al.*, 1980), that were detected before (McPherson *et al.*, 1963), simultaneous with (Eichner, 1956), or after the vulvar tumour (Diehl *et al.*, 1951). While these reports are intriguing, they were not derived from a defined population, nor were the cases compared to a control group. Using data on cancer incidence rates from the Connecticut Tumor Registry, Schoenberg (1977) showed that women who had cervical cancer were at greater risk for other anogenital malignancies compared with other women. In a hospital-based, case-control study of vulvar cancer, Mabuchi *et al.* (1985) found that six of 149 women with vulvar cancer but none of 149 controls had a history of prior urogenital cancer.

As part of a case-control study of women with *in situ* and invasive vulvar cancer, we had the opportunity to examine the hypothesis that multiple anogenital malignancies occur more commonly that would be predicted by chance.

### Patients and methods

With the assistance of the population-based cancer reporting system in western Washington, we identified all women ages 18–79 years with *in situ* or invasive vulvar cancer who were residents of King, Pierce, or Snohomish Counties and were diagnosed during 1980–1985. All diagnoses were histologically confirmed. Women were interviewed during 1984–1987, after being approached by means of a letter from their physicians. The response status of women with cancer (all histologies) is shown in Table I. Only women with squamous cell carcinoma of the vulva (91% of all interviewed cases) comprised the cases in this analysis. Among the interviewed cases, a total of 123 women with *in situ* vulvar cancer and a total of 35 women with invasive vulvar cancer met our eligibility criteria.

Two separate control groups were selected: (1) women with certain benign conditions of the vulva and (2) a random sample of the population obtained by random digit dialing. All hospital pathology departments and the major independent pathology laboratories were contacted for permission to abstract the names, ages, biopsy dates, and physicians for women who were biopsied for benign vulvar conditions. Virtually all (97%) of the pathology laboratories agreed to participate. Only those diagnoses thought to be unrelated to vulvar cancer and to condyloma acuminatum were abstracted and used to select women for the 'biopsy' control group. The majority of the 113 women in the 'biopsy' control group had a diagnosis of nevi (28), inflammation (12), epidermoid cyst (12), lentigo (10), fibroepithelial polyp (8), skin tag (7), hydradenoma (6), abscess (4), or haemangioma (3). The remaining 23 women in this control group had one of 17 other diagnoses. Among 104 women who reported why they visited a physician at the time the biopsy was performed, most of the women stated that they visited the physician for a routine exam or a pregnancy-related exam (45) or because they were concerned about the condition that was biopsied during their visit (33). Other reasons women gave for that physician visit included complaints associated with bleeding (7), other gynaecologic problems (8), non-gynaecologic problems (6), and surgery for non-gynaecologic conditions (5). This control group was selected originally to provide tissue controls for another aspect of this study. An attempt was made to match a 'biopsy' control to a case on the basis of diagnosis year, county of residence, and age (5-year groups). Although we were able to match on year and age, we found it difficult to find matches for women from the two less urban counties in our study and as a result, we did not find a match for 45 women. The controls we could identify were also approached by means of a letter from their physician. Their response status is shown in Table I. Most non-respondents were lost to follow-up because they did not obtain regular care from the physician who performed the biopsy and, often, so little information about them was available that public records could not be used successfully to trace them.

Using the method of Waksberg (1978), random digit dialing was used to obtain the second control group, i.e., a

Correspondence: K.J. Sherman.
Received 12 October 1987; and in revised form 18 January, 1988.

sample of women from the population at large. These women were frequency-matched to cases on county of residence and age (5-year groups). A total of 2,571 telephone numbers were dialed. For 3% of the phone numbers called, we were unable to determine if they were residential or non-residential. A household census was successfully conducted for 95% of the residences called. Among the eligible women for the study, 76% were interviewed.

The interview that was administered to both cases and controls was structured and was conducted in person. It included questions about sexual, reproductive, contraceptive, and medical histories (including other cancers and sexually transmitted diseases), demographic characteristics, Pap smear history, and the use of tobacco, alcohol, and social drugs. All information was collected only up to the date of diagnosis of the case or a comparable time period for her control. Women were asked about a history of all cancers, including the anatomic site, age at diagnosis, type of treatment, physician treating the cancer and the geographic area where the diagnosis was made. For the purpose of these analyses, diagnoses of prior or concurrent malignancy were divided into two groups: anogenital cancers (cancers of the cervix, vagina, anus) and other cancers. An earlier or concurrent vulvar cancer was not included as an anogenital cancer (no controls reported a history of vulvar cancer). For some analyses, anogenital cancers were further classified into concurrent tumours, e.g., those tumours diagnosed within one year of the date the vulvar cancer was diagnosed (or the vulvar biopsy was taken) and prior tumours, those tumours diagnosed more than one year before the vulvar cancer diagnosis.

Twelve women reported a history of uterine cancer. Eight of these tumours were noted as either cervical or endometrial in the Tumour Registry abstract, and this information was used to classify these tumours as anogenital or other. Because three of the four remaining women stated that their tumour occurred before they were 30, it is likely that these tumours were of the cervix, and thus should be considered anogenital cancers. Several analyses were conducted classifying these tumours as other, and the results were not materially altered.

Relative risks were approximated by the odds ratio for which the conditional maximum likelihood estimate was computed. Exact confidence limits were calculated using the algorithm of Mehta *et al.* (1986). Because all women with invasive vulvar cancer were over 35 years of age, this group of cases was compared only to those controls over the age of 35.

An attempt was made to assess the accuracy of a subject's reported history of prior or concurrent cancer by examining the records of the population-based SEER Tumour Registry. These records include all individuals who had a diagnosis of cancer (except basal and squamous cell skin cancer) in 13 counties of western Washington after 1973. In addition, tumours diagnosed outside the geographic boundaries of the Tumour Registry or before it began are recorded on the Registry abstracts of cancer cases whenever this information is available.

Twenty-nine of the 33 anogenital tumours that were reported by the cases and three of seven reported by the controls conformed to the conditions that were necessary for verification using the records of the Tumour Registry. Of those, 27 of 29 of the cases' tumours and two of three of the controls' neoplasms had been identified in the records as well. On the other hand, two cases and one RDD control had prior tumours identified in the Registry records that they did not report during the interview. For the purposes of these analyses, only cancers reported by the subjects were included.

### Results

Table II presents some demographic characteristics of cases and controls. Women with invasive vulvar cancer tended to be older than those with *in situ* vulvar cancer. The age distributions of the biopsy and RDD controls were more similar to those of the women with *in situ* vulvar cancer. On the average, cases reported less education and lower income than either of the control groups. Biopsy controls were more likely to have resided in King County than were cases or RDD controls. Cases and controls were similarly distributed in terms of race.

**Table I** Response status of women with vulvar cancer and biopsy controls

| | Cases[a] | | Controls | |
|---|---|---|---|---|
| *Response* | *No.* | % | *No.* | % |
| Interviewed | 174 | 67.2 | 113 | 59.8 |
| Deceased | 20 | 7.7 | 6 | 3.2 |
| Physician refusal | 8 | 3.1 | 10 | 5.3 |
| Patient refusal | 38 | 14.7 | 18 | 9.5 |
| Too ill | 4 | 1.5 | 5 | 2.7 |
| Lost to follow up | 15 | 5.8 | 37 | 19.6 |

[a]Only 158 interviewed cases had squamous cell carcinoma of the vulva, and the analyses included only these cases.

Women with *in situ* vulvar cancer reported a history of at least one primary anogenital cancer (three of these cases reported two anogenital cancers) at or before the reference date more often than women in either control group (age-adjusted OR = 6.2, 95% CI = 2.3–21.4, biopsy controls as comparison; OR = 29.8, 95% CI = 7.3–263.4, RDD controls as comparison, (Table III). Women with invasive vulvar cancer had a similar excess (age-adjusted OR = 3.5, 95% CI = 0.7–20.6, biopsy controls as comparison; OR = 19.1, 95% CI = 2.7–228.9, RDD controls as comparison, (Table III). Strong associations were present when prior and concurrent tumours were examined separately (Table III). Among the 19 women who were diagnosed with *in situ* vulvar cancer who reported a prior anogenital tumour, an average of 12 years elapsed between the diagnosis of the anogenital and the vulvar tumours. Only 16% of these women reported an interval of less than 5 years between the two diagnoses, and 42% of the women reported an interval of more than 10 years between tumours. All four women who were diagnosed with invasive vulvar cancer and who reported a prior anogenital tumour reported an interval of more than 10 years between tumours. The majority (81%) of these non-vulvar anogenital cancers were cervical tumours, but there were also five anal and two vaginal cancers reported.

These associations were not explained by case-control differences in demographic characteristics. Adjusting separately for education, income, reference year, and county of residence altered the odds ratio only slightly. Because only five biopsy controls and two RDD controls reported a history of anogenital cancer, adjusting simultaneously for all demographic differences between cases and controls was not feasible.

The association between vulvar cancer and prior anogenital tumours did not appear to be a consequence of radiation treatment of the initial anogenital tumour. Only four women reported treatment of their initial tumour by radiation, and the magnitude of the association was unaffected by the exclusion of these women.

Over 99% of all cases and controls reported having had a Pap smear at least once. After adjusting for the average interval between Pap smears during the ten years before reference date (more than 5 years, between 1 and 5 years, 1 year or less), a strong association remained.

By contrast, there was no association between *in situ* or invasive vulvar cancer and a history of prior or concurrent tumours of other than anogenital sites (Table III). The

**Table II** Demographic characteristics of cases and controls

| | *In situ* | | *Invasive* | | *Biopsy controls* | | *RDD controls* | |
|---|---|---|---|---|---|---|---|---|
| | *N=123* | % | *N=35* | % | *N=113* | % | *N=212* | % |
| *Age (years)* | | | | | | | | |
| <40 | 52 | 42.3 | 3 | 8.6 | 44 | 38.9 | 76 | 35.8 |
| 40–59 | 39 | 31.7 | 9 | 25.7 | 38 | 33.6 | 74 | 34.9 |
| 60+ | 32 | 26.0 | 23 | 65.7 | 31 | 27.4 | 62 | 29.2 |
| *Education*[c] | | | | | | | | |
| <High school | 71 | 58.8 | 24 | 56.6 | 42 | 37.7 | 83 | 39.1 |
| >High school | 52 | 41.1 | 11 | 43.3 | 71 | 62.2 | 129 | 60.9 |
| *Annual family income*[a,c] | | | | | | | | |
| <$15,000 | 54 | 44.9 | 17 | 42.3 | 28 | 25.6 | 58 | 27.6 |
| $15,000–30,000 | 44 | 35.6 | 7 | 15.4 | 42 | 37.1 | 84 | 40.0 |
| >$30,000 | 24 | 19.4 | 9 | 37.2 | 41 | 36.9 | 68 | 32.4 |
| *County of residence*[c] | | | | | | | | |
| King | 81 | 66.1 | 21 | 61.1 | 94 | 83.3 | 123 | 58.0 |
| Pierce | 20 | 16.6 | 8 | 17.9 | 12 | 10.7 | 51 | 24.1 |
| Snohomish | 22 | 17.3 | 6 | 20.9 | 7 | 6.0 | 38 | 17.9 |
| *Race*[c] | | | | | | | | |
| White | 119 | 96.5 | 32 | 93.5 | 109 | 96.6 | 194 | 91.5 |
| Non-white | 4 | 3.6 | 3 | 6.4 | 4 | 3.3 | 18 | 8.5 |
| *Frequency of prior Pap smear*[b,c] (within last 10 years) | | | | | | | | |
| <5 years | 20 | 17.7 | 13 | 34.9 | 14 | 12.9 | 41 | 19.4 |
| 2–5 years | 20 | 15.9 | 6 | 20.9 | 28 | 24.8 | 57 | 27.0 |
| At least yearly | 82 | 66.3 | 16 | 44.0 | 71 | 62.1 | 113 | 53.6 |

[a]One *in situ* case, 1 invasive case, 2 biopsy controls and 2 RDD controls refused to answer this question. One invasive case did not know the answer to this question; [b]One *in situ* case and one RDD control did not know the answer to this question; [c]All of these percentages are adjusted to the age distribution of the RDD controls (18–39, 40–59, 60–79).

**Table III** Association between vulvar cancer and other primary tumours in women[a]

| | *In situ N=123* | *Invasive N=35* | *Biopsy N=113* | *RDD N=212* | *In situ/ Biopsy RR 95% CI* | *In situ/ RDD RR 95% CI* | *Invasive/ Biopsy RR 95% CI* | *Invasive/ RDD RR 95% CI* |
|---|---|---|---|---|---|---|---|---|
| All with prior and/or current anogenital cancers | 28 22% | 5 24.8% | 5 4.6% | 2 1% | 6.2<br>2.3–21.4 | 29.8<br>7.3–263.2 | 3.5<br>0.7–20.6 | 19.1<br>2.7–228.9 |
| Site:[b] | | | | | | | | |
| Cervical | 24 18.8% | 5 24.8% | 4 3.6% | 2 1% | 6.5<br>2.1–26.5 | 24.6<br>5.9–281.5 | 5.4<br>0.9–40.3 | 19.1<br>2.7–228.9 |
| Other | 7 5.4% | 0 0% | 1 0.9% | 0 0% | 6.7<br>0.8–307.3 | ∞<br>3.2–∞ | –<br>– | –<br>– |
| Timing:[b] | | | | | | | | |
| Prior | 19 15.4% | 4 12.9% | 3 2.8% | 2 1% | 6.6<br>1.9–36.0 | 19.0<br>4.4–171.0 | 3.8<br>0.6–29.6 | 13.3<br>1.7–166.3 |
| Concurrent | 11 8.2% | 1 11.9% | 2 1.8% | 0 0% | 5.3<br>1.1–50.6 | ∞<br>5.5–∞ | 2.4<br>0.03–216.1 | ∞<br>0.32–∞ |
| All with prior and/or concurrent non-anogenital cancers | 7 6.4% | 4 7.8% | 7 6.5% | 17 8.0% | 0.97<br>0.3–3.5 | 0.7<br>0.2–1.8 | 1.0<br>0.2–4.5 | 0.9<br>0.2–3.0 |

[a]All percentages are age-adjusted to the distribution of the RDD population; all RRs are age-adjusted; [b]Two *in situ* cases reported one prior and one concurrent anogenital cancer; one *in situ* case reported two concurrent anogenital cancers.

majority of the other cancers were of the breast (N=13), skin (N=11), endometrium (N=5), and colon (N=2).

### Discussion

We found that women with *in situ* or invasive vulvar cancer reported a history of prior or concurrent anogenital tumours far more often than did women in either control group. This association is likely to be confined to squamous cell tumours. Of the 16 women with non-squamous cell vulvar tumours (these diagnoses included melanoma, basal cell cancer, and Paget's disease), none reported a prior or concurrent anogenital malignancy. The strong association of anogenital tumours and squamous cell vulvar cancer, when contrasted with the observation of no excess risk of non-anogenital tumours among women in the case groups, suggests that these findings are real.

Nonetheless, several biases that might have produced a spurious result must be considered. First, we encountered a moderate level of nonresponse. However, we have reason to believe that the observed association did not result from the non-interviewed cases having an unusually low frequency of prior or concurrent anogenital tumours. Using the Tumour Registry records to identify prior or concurrent anogenital cancers among all eligible cases of vulvar cancer, we found that actually a slightly higher percentage of non-interviewed than interviewed cases (23% vs 19%) had had such tumours.

Second, the excess number of anogenital tumours observed in cases conceivably occurred as a result of more intensive follow-up of women with cervical cancer leading to an increased likelihood of detecting inconspicuous vulvar lesions in these women. However, since women with invasive vulvar cancer, a disease that rarely remains undetected for more than a short period of time, reported a history of prior or concurrent anogenital cancer more often than controls, although less commonly than women with *in situ* vulvar cancer, such a detection bias is unlikely to completely account for the association in comparisons involving this group of cases. In addition, the biopsy controls also had a vulvar biopsy at reference date and thus, surveillance for the case groups and this control group should be comparable. The lower risk estimates we found when using the biopsy controls as a comparison group could well have been due to the differences in surveillance among the women in this control group and the women in the random digit dialed control group. On the other hand, some of the biopsy controls may have been biopsied because they had a prior history of conditions that can be precursors to vulvar cancer, a possibility that, if true, would spuriously lower the risk estimates.

As noted earlier, cases and controls did not differ substantially in their accuracy of reporting a history of prior or concurrent anogenital cancer; thus, recall bias could not have explained the higher proportion of anogenital cancers among the cases. Nor were differences in the frequency of cervical screening among cases and controls responsible for the positive association of anogenital cancers with vulvar cancer, because the association remained after adjustment for differences in screening history.

We believe that the most reasonable interpretation of these observations is that the association is real. However, our results do not allow us to discriminate between several possibilities regarding its basis. One possibility is that the association between vulvar cancer and a history of prior or concurrent anogenital cancer reflects a common aetiology, which is likely to be a sexually transmitted disease. Or, it could be that these tumours are caused by different sexually transmitted diseases, several of which often occur in the same individual. Indeed, it is well known that people infected with one sexually transmitted organism are more likely to be at risk for other sexually transmitted infections. And, evidence of exposure to many sexually transmitted organisms has been observed in women with cervical cancer, including syphilis, genital herpes, human papilloma virus, and *Chlamydia* (Levin *et al.*, 1942; Rojel, 1952; Melnick *et al.*, 1974; Kauffman & Adam, 1986; Durst *et al.*, 1983; Syrjanen *et al.*, 1985; Schachter *et al.*, 1982; Cardillo, 1985). These same diseases also have been found more often in women with vulvar cancer than would be expected in a similar group of women without this disease (Franklin & Rutledge, 1974; Mabuchi *et al.*, 1985; Schwartz & Naftolin, 1981; Rueda-Leverone *et al.*, 1987) and have been found in patients with anal cancer more often than in controls (Gal *et al.*, 1987; Bogomoletz *et al.*, 1985; Daling *et al.*, 1987).

Recently, there has accumulated strong evidence to suggest that human papilloma virus is one aetiology common to all anogenital cancers. Human papilloma virus has been found in tumour tissue from the cervix, vulva, vagina, and anus (Okagaki, 1984; Durst *et al.*, 1983; Syrjanen *et al.*, 1985; Rueda-Leverone *et al.*, 1987; Gal *et al.*, 1987; Winkler & Richart, 1987). In addition, there have been several reports of multicentric human papilloma virus infections in the same individual (Sfameni *et al.*, 1986; Walker *et al.*, 1983; Bergeron *et al.*, 1987). Finally, there have been several reports of finding the same type of human papilloma virus in multiple anogenital cancers in the same individual (Weed *et al.*, 1983; McCance *et al.*, 1985; Beckmann *et al.*, 1988). Genital herpes and smoking are two additional putative risk factors that are found more often among cases with cervical (Clarke *et al.*, 1982; Brinton *et al.*, 1986), anal (Daling *et al.*, 1987), and vulvar (Schwartz & Naftolin, 1981; Newcomb *et al.*, 1984) cancer than among controls. Whether herpes simplex virus type 2, human papilloma virus, and smoking are independent risk factors or whether they interact with each other in the development of anogenital cancers remains to be clarified.

This work was supported in part by Grant No. CA 35881 from the National Cancer Institute.

### References

BECKMANN, A.M., KIVIAT, N.B., DALING, J.R., SHERMAN, K.J. & McDOUGALL, J.K. (1988). Human papillomavirus type 16 in multifocal cancers of the female genital tract. *Int. J. Gynecol. Pathol.*, **7**, 39.

BERGERON, C., FERENCZY, A., SHAH, K.V. & NAGHASHFAR Z. (1987). Multicentric human papillomavirus infections of the female genital tract: correlation of viral types with abnormal mitotic figures, colposcopic presentation, and location. *Obstet. Gynecol.*, **69**, 736.

BOGOMOLETZ, W.V., POTET, F. & MOLAS, G. (1985). Condylomata acuminata and verrucous squamous carcinoma of the perianal and anorectal region: a continuous precancerous spectrum? *Histopathology*, **9**, 1155.

BRINTON, L.A., SCHAIRER, C., HAENSZEL, W. & 4 others (1986). Smoking and invasive cervical cancer. *J. Am. Med. Assoc.*, **255**, 3265.

CABERRA, A., TSUKADA, Y., PICKREN, J.W., MOORE, R. & BROSS, I.D.J. (1966). Development of lower genital tract carcinomas in patients with anal carcinoma. *Cancer*, **19**, 470.

CARDILLO, M.R. (1985). Association of human papilloma virus and *Chlamydia trachomatis* infections with incidence cervical neoplasia. *Eur. J. Gynecol. Oncol.*, **6**, 218.

CLARKE, E.A., MORGAN, R.W. & NEWMAN, A.M. (1982). Smoking as a risk factor in cancer of the cervix: additional evidence from a case-control study. *Am. J. Epidemiol.*, **115**, 59.

CROMER, J.K. (1963). Further observations on the multicentric origin of carcinomas of the female anogenital tract. *Am. Surg.*, **29**, 793.

DALING, J.R., WEISS, N.S., HISLOP, T.G. & 7 others (1987). Sexual practices, sexually transmitted diseases and the incidence of anal cancer. *New Engl. J. Med.*, **31**, 973.

DAY, J.C. (1958). Second primary malignant tumor in gynecology. *Am. J. Obstet. Gynecol.*, **75**, 976.

DIEHL, W.K., BAGGETT, J.W. & SHELL, J.H. (1951). Vulvar cancer. *Am. J. Obstet. Gynecol.*, **62**, 1209.

DURST, M., GISSMAN, L., IKENBERG, H. & ZUR HAUSEN H. (1983). A papillomavirus DNA from a cervical carcinoma and its prevalence in cancer biopsy samples from different geographic regions. *Proc. Natl Acad. Sci. USA.*, **80**, 3812.

EICHNER, E. (1956). Multiple carcinoma *in situ*. *Obstet. Gynecol*, **8**, 508.

FRANKLIN, E.W. & RUTLEDGE, F.D. (1974). Epidemiology of epidermoid carcinoma of the vulva. *Obstet. Gynecol.*, **39**, 165.

FRIEDRICH, E.G., WILKINSON, E.J. & FU, Y.S. (1980). Carcinoma *in situ* of the vulva: a continuing challenge. *Am. J. Obstet. Gynecol.*, **136**, 830.

GAL, A.A., MEYER, P.R. & TAYLOR, C.R. (1987). Papillomavirus antigens in anorectal condyloma and carcinoma in homosexual men. *J. Am. Med. Assoc.*, **257**, 337.

KAUFFMAN, R.H. & ADAM, E. (1986). Herpes simplex virus and human papillomavirus in the development of cervical carcinoma. *Clin. Obstet. Gynecol.*, **29**, 678.

LEVIN, M.L., KRESS, L.C. & GOLDSTEIN, H. (1942). Syphilis and cancer. *NY State J. Med.*, **42**, 1737.

MABUCHI, K., BROSS, D.S. & KESSLER, I.I. (1985). Epidemiology of cancer of the vulva. *Cancer*, **55**, 1843.

McCANCE, D.J., CLARKSON, P.K., DYSON, J.L., WALKER, P.G. & SINGER, A. (1985). Human papillomavirus types 6 and 16 in multifocal intraepithelial neoplasias of the female lower genital tract. *Br. J. Obstet. Gynecol.*, **92**, 1093.

McPHERSON, H.A., DIDDLE, A.W., GARDNER, W.H. & WILLIAMSON, P.J. (1963). Epidermoid carcinoma of the cervix, vagina, and vulva: a regional disease. *Obstet. Gynecol.*, **21**, 145.

MEHTA, C.R., PATEL, N.R. & GRAY, R. (1986). Computing an exact confidence interval for the common odds ratio in several $2 \times 2$ contingency tables. *J. Am. Stat. Assoc.*, **80**, 969.

MELNICK J.L., ADAM, E. & RAWLS, W.E. (1974). The causative role of herpesvirus type 2 in cervical cancer. *Cancer*, **34**, 1375.

NEWCOMB, P.A., WEISS, N.S. & DALING, J.R. (1984). Incidence of vulvar carcinoma in relation to menstrual, reproductive, and medical factors. *J. Natl Cancer Inst.*, **73**, 391.

OKAGAKI, T. (1984). Female genital tumors associated with human papillomavirus infection and the concept of genital neoplasm–papilloma syndrome (GENPS). *Pathol. Ann.*, **19**, 31.

PETERS, R.K., MACK, T.M. & BERNSTEIN, L. (1984). Parallels in the epidemiology of selected anogenital carcinomas. *J. Natl Cancer Inst.*, **72**, 609.

ROJEL, J. (1952). The interrelation between uterine cancer and syphilis. *Acta. Path. Microbiol. Scand. Suppl.*, **92**, 68.

RUEDA-LEVERONE, N.G., DIPAOLA, G.R., MEISS, R.P., GRACIELA VIGHI, S. & LLAMOSAS, F. (1987). Association of human papillomavirus infection and vulvar intraepithelial neoplasia: a morphological and immunohistochemical study of 30 cases. *Gynecol. Oncol.*, **26**, 331.

SCHACHTER, J., HILL, E.C., KING, E.B. & 4 others (1982). *Chlamydia trachomatis* and cervical neoplasia. *J. Natl Cancer Inst.*, **248**, 2134.

SCHOENBERG, B.S. (1977). Multiple primary malignant neoplasms: the Connecticut experience 1935–1964. Recent results. *Cancer Res.*, **58**, 1.

SCHWARTZ, P.E. & NAFTOLIN, F. (1981). Type 2 herpes simplex virus and vulvar carcinoma *in situ*. *New Engl. J. Med.*, **205**, 517.

SFAMENI, F.S., OSTOR, A.G., CHANEN, W. & FORTUNE, D.W. (1986). The association between vulvar condylomata acuminata, cervical wart virus infection, and cervical intraepithelial neoplasia. *Aust. NZ J. Obstet. Gynecol.*, **26**, 149.

SYRJANEN, K., DEVILLERS, E.M., SAARIKOSKI, S. & 4 others (1985). Cervical papillomavirus infection progressing to invasive cancer in less than three years. *Lancet*, **i**, 510.

TAUSSIG, F.L. (1940). Cancer of the vulva: analysis of 155 cases (1911-1940). *Am. J. Obstet. Gynecol.*, **40**, 764.

WAKSBERG, J. (1978). Sampling methods for random digit dialing. *J. Am. Stat. Assoc.*, **77**, 40.

WALKER, P.G., COLLEY, N.V., GRUBB, C., TEJERINA, A. & ORIEL, J.D. (1983). Abnormalities of the uterine cervix in women with vulvar warts. *Br. J. Vener. Dis.*, **59**, 120.

WEED, J.C., LOZIER, C. & DANIEL, S.C. (1983). Human papillomavirus in multifocal invasive female genital tract malignancy. *Obstet. Gynecol., Suppl.*, **62**, 83S.

WINKLER, B. & RICHART, R.M. (1987). Human papillomavirus and gynecologic neoplasia. *Curr. Probl. Obstet. Gynecol. Fertil.*, **10**, 49.